# Nuclear Respiratory Factor-1, a Novel SMAD4 Binding Protein, Represses TGF-β/SMAD4 Signaling by Functioning as a Transcriptional Cofactor

**DOI:** 10.3390/ijms22115595

**Published:** 2021-05-25

**Authors:** Nirmal Rajasekaran, Kyoung Song, Jin-Hee Lee, Yun Wei, Özgür Cem Erkin, Hunseok Lee, Young-Kee Shin

**Affiliations:** 1 Laboratory of Molecular Pathology and Cancer Genomics, Research Institute of Pharmaceutical Sciences and College of Pharmacy, Seoul National University, Seoul 08826, Korea; nirmalpharma@gmail.com (N.R.); jinheelee80@gmail.com (J.-H.L.); yunwei.bio@gmail.com (Y.W.); ryanlee0@snu.ac.kr (H.L.); 2 College of Pharmacy, Duksung Women’s University, Seoul 01369, Korea; songseoul17@duksung.ac.kr; 3 Department of Bioengineering, Faculty of Engineering, Adana Science and Technology, Adana 01250, Turkey; ocerkin@yahoo.com; 4 Department of Molecular Medicine and Biopharmaceutical Sciences, Graduate School of Convergence Science and Technology, Seoul National University, Seoul 08826, Korea; 5 Bio-MAX, Seoul National University, Seoul 08826, Korea

**Keywords:** NRF1, SMAD4, transforming growth factor-β, p15INK4b, tumor suppression

## Abstract

SMAD4, a key regulator of transforming growth factor-β (TGF-β) signaling, plays a major role in cell growth, migration, and apoptosis. In particular, TGF-β/SMAD induces growth arrest, and SMAD4 induces the expression of target genes such as *p21WAF1* and *p15INK4b* through its interaction with several cofactors. Thus, inactivating mutations or the homozygous deletion of *SMAD4* could be related to tumorigenesis or malignancy progression. However, in some cancer types, *SMAD4* is neither mutated nor deleted. In the current study, we demonstrate that TGF-β signaling with a preserved SMAD4 function can contribute to cancer through associations with negative pathway regulators. We found that nuclear respiratory factor-1 (NRF1) is a novel interaction SMAD4 partner that inhibits TGF-β/SMAD4-induced *p15INK4b* mRNA expression by binding to SMAD4. Furthermore, we confirmed that NRF1 directly binds to the core region of the *SMAD4* promoter, thereby decreasing *SMAD4* mRNA expression. On the whole, our data suggest that NRF1 is a negative regulator of SMAD4 and can interfere with TGF-β/SMAD-induced tumor suppression. Our findings provide a novel perception into the molecular basis of TGF-β/SMAD4-signaling suppression in tumorigenesis.

## 1. Introduction

In vertebrates, the transforming growth factor-β (TGF-β) pathway regulates the expansion of epithelial and neural tissues and the immune system and functions in wound repair. As a consequence, malfunctions in TGF-β pathway signaling often result in tumorigenesis [1]. In the canonical TGF-β signaling pathway, exogenous signaling molecules (ligands) activate the plasma membrane-bound serine/threonine kinase receptors, which further activate the pathway via intracellular SMAD mediators. The TGF-β receptor complex phosphorylates the transcription factors SMAD2 and SMAD3, which then bind to SMAD4, translocate into the nucleus, and associate with diverse DNA-binding cofactors to target genes for regulation. SMAD4, a key downstream component in this system, was first isolated as a tumor-suppressor gene in human pancreatic ductal carcinomas [2,3]. The homozygous deletion or inactivating mutations of SMAD4 play a crucial role in the malignant progression of certain cancer types [4,5]. By contrast, SMAD4 mutations are rarely observed in other cancer types. The function and significance of positive or negative regulators of SMAD4 are being studied in many cancers [6].

Nuclear respiratory factor 1 (NRF1) is a transcription factor that regulates a myriad of cellular functions, including mitochondrial biogenesis, DNA replication and repair, proliferation, and apoptosis. NRF1 was originally identified as a cytochrome-c activator [7]. NRF1 and NRF2 regulate a multitude of genes essential for the expression of proteins implicated in mitochondrial functions and biogenesis [8], mitochondrial replication, gene expression, and protein import and assembly [9,10,11,12]. Besides its role in the regulation of mitochondrial functions, NRF1 is also a crucial player in histone gene expression and acts as a regulator of cell growth and proliferation [13,14]. Accumulating evidences also implicate that NRF1 expression and its transcription factor activity may contribute to the pathogenesis of breast cancer, glioblastoma, and neuronal dysfunction [15,16,17].

Modulation of the transcription factor function through protein–protein interactions is a crucial process in the activation or repression of signal transduction pathways [1,5]. NRF1 binds to the promoter region of E2F downstream signaling molecules, modulating their transcription and thereby controlling cell cycle progression [13]. The interaction between NRF1 and other cofactors is also presumed to have a dramatic and diverse effect in different cell types. NRF1 transmits extracellular physiological changes in a tissue-specific manner via its interaction with the PGC-1 family members [18,19,20]. Employing a computational analysis to predict the tissue-specific combinatorial gene regulation, Yu et al. identified NRF1, SMAD3, and E2F as the top three signaling hubs for interactions between transcription factors in the cervix [21].

Here, we demonstrate that NRF1 is a novel SMAD4-binding partner and that the interaction between SMAD4 and NRF1 can repress TGF-β/SMAD4–induced tumor-suppressor functions. Besides, NRF1 can function as a transcription factor and influence SMAD4 expression. We also identified p15INK4b, a cell cycle inhibitor, as an important target for TGF-β/SMAD4-induced tumor-suppressor functions. These results suggest a possible tumorigenic role for NRF1 and may also explain the paradoxical lack of SMAD4 mutations in some types of cancers.

## 2. Results

### 2.1. NRF1 Is a Novel SMAD4-Binding Partner

**Figure 1:**
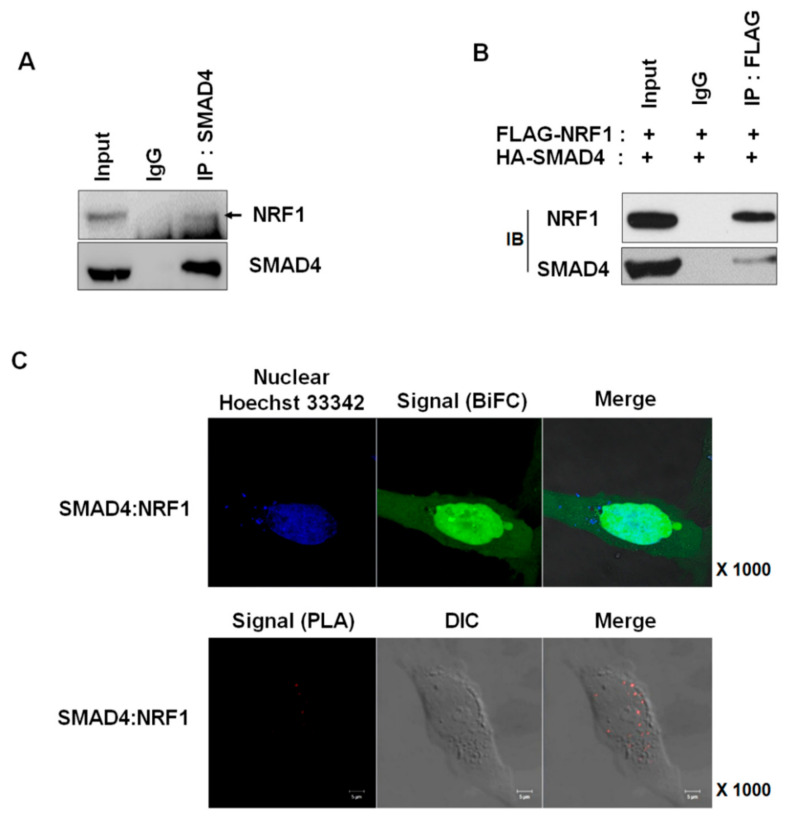
Identification of NRF1 as a SMAD4-interacting protein. (**A**) Endogenous and (**B**) exogenous SMAD4 interact with NRF1. HeLa cell lysates were immunoprecipitated with the indicated antibody or IgG. (**C**) Visualization of SMAD4 and NRF1 interactions using the BiFC analysis (upper panel) and in situ PLA assay (lower panel). For the BiFC analysis, HA-SMAD4-VC155 and FLAG-NRF1-VN173 constructs were co-transfected into HeLa cells. After 24 h, bound proteins were detected by visualizing the fluorescence signal. For in situ PLA assays, cultured HeLa cells were fixed and incubated with NRF1 or SMAD4 antibodies and the PLA probe. Fluorescence was imaged with a confocal microscope system. DIC: differential interference contrast. Scale bar, 5 µm.

To explore SMAD4 regulation, we searched for SMAD4-binding partners using a baculovirus ProtoArray; the screen identified NRF1 and other novel interaction proteins (Table 1). Both endogenously and exogenously expressed SMAD4 and NRF1 interacted in vitro, as confirmed by immunoprecipitation (Figure 1A,B). We also assessed NRF1 and SMAD4 binding using a BiFC assay [22,23] and in situ PLA to exclude the possibility of binding artifacts (Figure 1C). The BiFC assay not only detects protein–protein interactions in living cells but also identifies the intracellular location where these interactions occur. The plasmids used for the BiFC assay were constructed as previously described [24] and were transfected into HeLa cells. The binding of NRF1to SMAD4 was observed, particularly within the nucleus. In situ PLA, like BiFC, not only confirms the cellular location of protein-binding interactions but can also confirm the binding patterns of proteins expressed at low levels and identify transient binding interactions. As with BiFC, the in situ PLA results demonstrated that SMAD4 and NRF1 interact within the nucleus. Our multiple experimental results confirm that NRF1 is a novel SMAD4-binding partner.

### 2.2. SMAD4 MH1 and MH2 Domains Interact with the NRF1 Dimerization Domain

**Figure 2:**
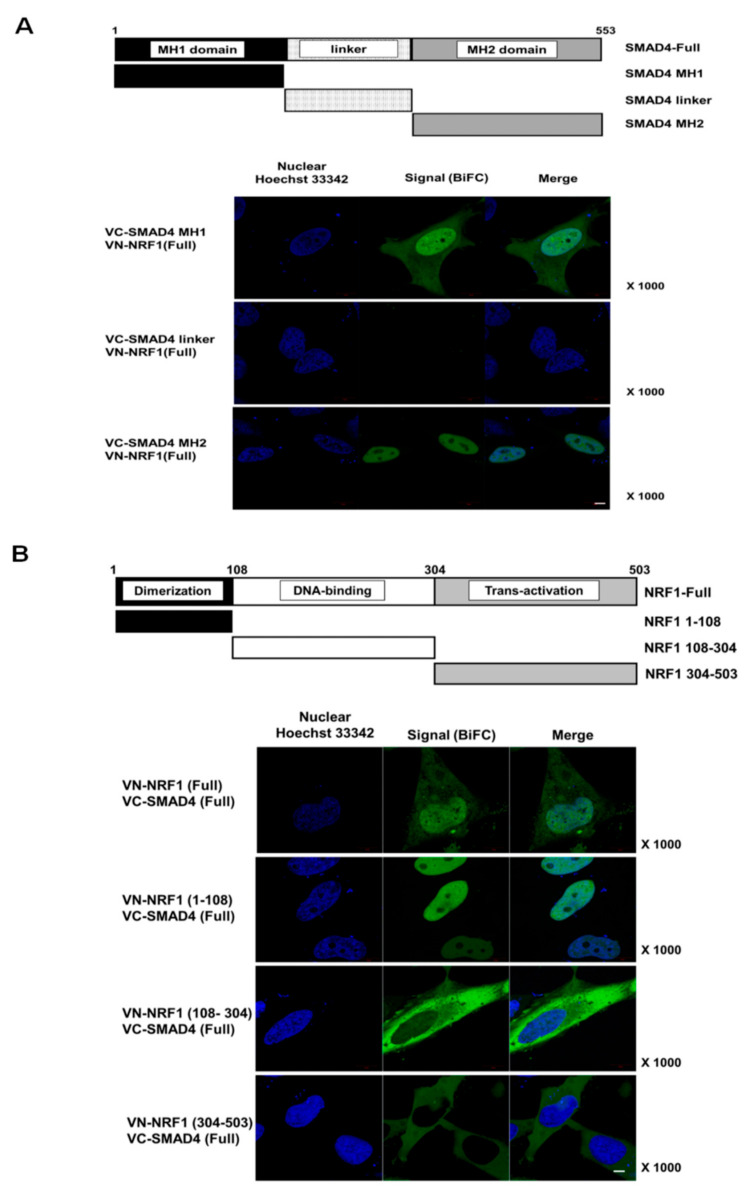
Interaction domains of SMAD4 and NRF1. (**A**) Scheme for the deletion constructs, according to the SMAD4 functional domains (upper panel) and identification of the binding between full-length (FL) NRF1 and SMAD4 truncations (SMAD4 MH1, MH2, and linker) using the BiFC analysis (lower panel). (**B**) Scheme for the deletion of constructs according to the NRF1 functional domains (upper panel), and the identification of binding between FL SMAD4 (1-503) and truncated (1-108, 108-304, and 304-503) NRF1 using the BiFC analysis (lower panel). Scale bar, 5 µm.

**Figure 3:**
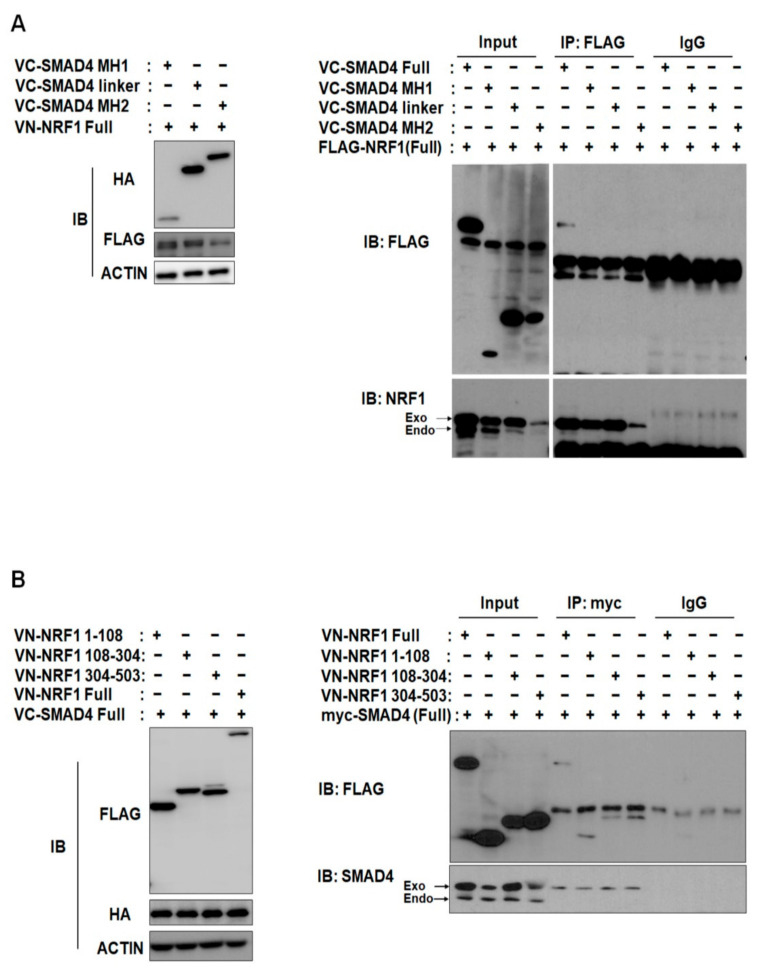
NRF1 interacts with SMAD4. (**A**) Exogenous NRF1 interacts with truncated SMAD4. Proteins were immunoprecipitated from HeLa cells with anti-FLAG, and Western blots were probed with the indicated antibodies. (**B**) Exogenous SMAD4 interacts with truncated NRF1. Proteins were immunoprecipitated from HeLa cells with anti-myc, and Western blots were probed with the indicated antibodies.

To identify the specific binding domains responsible for NRF1 and SMAD4 binding, we first generated six deletion constructs, each containing one functional domain of either NRF1 or SMAD4 (schematics in Figure 2A,B). Using the BiFC assay, we observed binding between full-length (FL) SMAD4 and each of the NRF1 deletion constructs and between FL NRF1 and each of the SMAD4 deletion constructs. Similar to WT, nuclear binding interactions were observed for SMAD4 MH1 and MH2 with FL NRF1 (Figure 2A) and for NRF1 (1–108) with FL SMAD4 (Figure 2A). Interestingly, the other NRF1 domain constructs (NRF1 (108–304) and NRF1 (304–503)) interacted with FL SMAD4 in the cytoplasm (Figure 2A). We observed cytoplasmic and nuclear binding for SMAD4 MH1 and FL NRF1, suggesting that NRF1 and SMAD4 could initially interact in the cytoplasm and then translocate into the nucleus. The binding specificity between SMAD4 and NRF1 was confirmed through BiFC competition assays with the transfection of tagged and nontagged proteins, in which nontagged NRF1 and SMAD4 were used as competitors (Supplementary Figure S1). The fluorescence signal from tagged NRF1 and SMAD4 was counted with and without the nontagged competitors, and the results revealed that each binding interaction was specific. NRF1 and SMAD4 binding was further confirmed by protein IP from HeLa cells (Figure 3A,B).

### 2.3. NRF1 Inhibits mRNA Expression of p15INK4b via SMAD4–NRF1 Interactions

**Figure 4:**
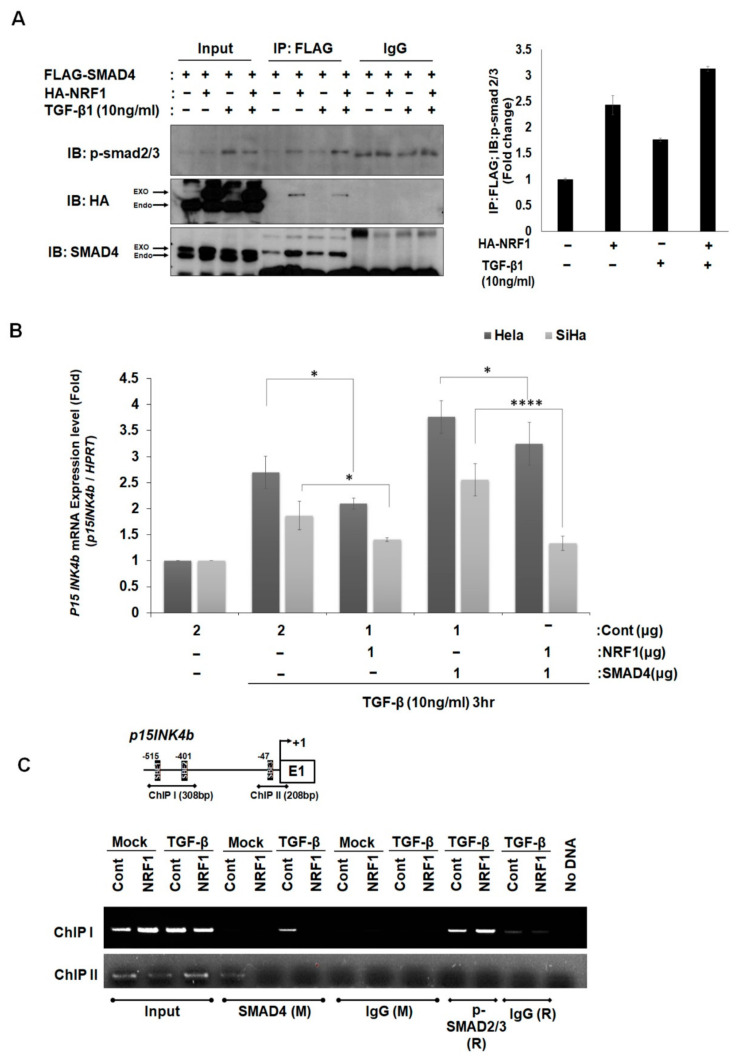
Repression of SMAD4-induced *p15INK4b* expression by NRF1. (**A**) Interactions between TGF-β-induced SMAD4 and p-SMAD2/3 are affected by NRF1 overexpression. FLAG-SMAD4 and HA-NRF1 plasmids were co-transfected into HeLa cells. After 24 h, the cells were treated with TGF-β (10 ng/mL) for 3 h. Proteins were immunoprecipitated from HeLa cells with anti-FLAG, and Western blots were probed with the indicated antibodies. (**B**) NRF1 inhibits TGF-β/SMAD4-induced *p15INK4b* mRNA expression. The qRT-PCR values were normalized to the housekeeping gene HPRT. The relative *p15INK4b* expression was calculated by comparing the test samples with the empty vector control. * *p* < 0.05 and **** *p* < 0.0001 (Student’s *t*-test). (**C**) ChIP assay for SMAD4 binding to the *p15INK4b* promoter either with or without NRF1. No DNA, negative control.

We hypothesized that NRF1 binding to SMAD4 influences the interaction between SMAD4 and SMAD2/3. As shown in Figure 4A, NRF1 overexpression slightly increased p-SMAD2/3 binding to SMAD4, even in the presence of TGF-β. We then asked whether NRF1 affects TGF-β-induced SMAD4 target gene expression. Using qRT-PCR, we quantified the transcription levels of a well-known SMAD4 target gene, *p15INK4b*, in HeLa and SiHa cells (Figure 4B). The qRT-PCR results showed that TGF-β/SMAD4-induced p15INK4b transcription was decreased when NRF1 was overexpressed in both HeLa (22.5%) and SiHa (24.5%) cells. Furthermore, we confirmed that the mRNA expression of *p15INK4b*, which was further increased by the overexpression of SMAD4, was dramatically inhibited by NRF1 in SiHa (47.5%) cells (Figure 4B).

We performed a ChIP analysis to confirm the observed effects of NRF1 on the direct interaction of SMAD4 to the SMAD-binding element (SBE) of the *p15INK4b* promoter in SiHa cells. SMAD4 directly binds to the two SBE regions (ChIP I and ChIP II) of the *p15INK4b* promoter, and this binding is inhibited by NRF1 overexpression (Figure 4C). DNA-pulldown assays support the ChIP assay results (Supplementary Figure S2). The NRF1-binding sites on the SMAD4 and *p15INK4b* promoters were mutated, and the binding of NRF1 was decreased in comparison to the wild-type promoter sequences. Taken together, the results suggest that, although the binding between SMAD4 and NRF1 moderately strengthened the interaction between SMAD4 and phosphorylated SMAD2/3, NRF1 inhibits TGF-β/SMAD4-induced *p15INK4b* mRNA expression by interfering with SMAD4 binding to the p15INK4b promoter, thus inhibiting the TGF-β/SMAD4-induced tumor-suppressor function.

### 2.4. NRF1 Regulates SMAD4 Expression through Its Function as a Transcription Factor

**Figure 5:**
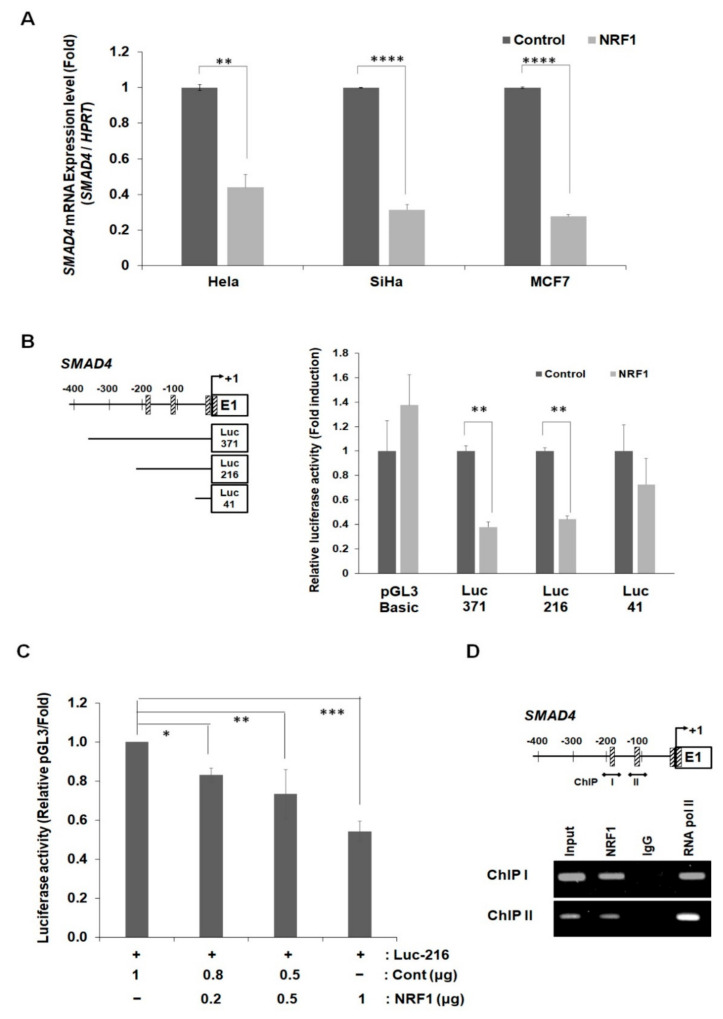
Inhibition of *SMAD4* mRNA expression by NRF1. (**A**) HeLa, SiHa, and MCF7 cells were transfected with NRF1 (2 μg) for 24 h, and mRNA transcription was analyzed using qRT-PCR. The qRT-PCR values were normalized to the housekeeping gene HPRT. ** *p* < 0.01 and **** *p* < 0.0001 (Student’s *t*-test). (**B**) Schematic of NRF1-binding elements in the *SMAD4* promoter and reporter constructs for the luciferase and ChIP assays (left panel). Rectangles with hash marks indicate NRF1-binding elements. Analysis of inhibition of the *SMAD4* promoter activity by NRF1 (right panel). Relative luciferase activity was normalized using the wild-type Rinella luciferase activity. Error bars represent the means ± SD. ** *p* < 0.01 (Student’s *t*-test). (**C**) Relative luciferase activity (dependent on NRF concentration). * *p* < 0.05, ** *p* < 0.01, ** *p* < 0.001 or **** *p* < 0.0001. (**D**) ChIP assay for NRF1 binding to the *SMAD4* promoter. RNA Pol II, positive control.

NRF1 is a well-known transcription factor that regulates the expression of a plethora of metabolic genes related to cellular growth and development. Hence, we examined whether NRF1 directly regulates SMAD4 transcription, in addition to the regulation we observed as a direct SMAD4-binding partner. Changes in the *SMAD4* mRNA expression, measured by qRT-PCR, were observed after NRF1 overexpression in HeLa, SiHa, and MCF7 cells (Figure 5A). The *SMAD4* mRNA levels were decreased by over 60% after NRF1 transfection in all three cell lines. We identified four putative NRF1-binding sites within the *SMAD4* promoter −500 bp to the transcription start site using MatInspector professional software and the internet-based TFSEARCH database (Supplementary Figure S3). We used a luciferase assay and generated three constructs, each containing a partial deletion mutant in the *SMAD4* promoter (Luc-371, -216, and -41), to identify the core region enabling NRF1-mediated transcriptional repression at the *SMAD4* promoter (Figure 5B, left). The transient co-transfection of NRF1 and each SMAD4 deletion mutant revealed changes in *SMAD4* promoter activity (Figure 5B, right). The 3′-truncated constructs Luc-216 and -41 were 30% and 60% inhibited, respectively. The inhibition of SMAD4 transcription by NRF1 was also confirmed using a dose-dependent NRF1 luciferase assay (Figure 5C). The inhibition of *SMAD4* promoter activity by NRF1 was concentration-dependent. Furthermore, the ChIP assay results confirmed that the two NRF1-binding elements (NBEs) are core sites for the regulation of SMAD4 expression by NRF1 (Figure 5D). NRF1 directly binds to the *SMAD4* promoter at ChIP sites I and II, confirming that NRF1 inhibits SMAD4 expression by directly binding to the *SMAD4* promoter. In addition to its effect on SMAD4 transcription, NRF1 binding to the *SMAD4* promoter also slightly inhibited the SMAD4 protein expression (Supplementary Figure S4).

## 3. Discussion and Conclusions

In this study, we found that the amino–terminal domain of NRF1 promotes NRF1 binding to SMAD4 in the nucleus based on multiple assays, including BiFC, in vivo proximity ligation assays, and protein immunoprecipitation from HeLa cells. Much to our surprise, the DNA-binding domain of NRF1, a domain necessary for its interaction with other binding partners [25,26], was not necessary for NRF1 and SMAD4 binding in the nucleus. The BiFC fluorescence signal detected for NRF1:SMAD4 binding may result from functional nuclear localization sequence 88–116 of SMAD4 MH1 and the SMAD4 MH2 domain mainly in the nucleus. A luciferase reporter assay was used to investigate the regulatory role of NRF1 as a transcription factor for SMAD4. Serial *SMAD4* promoter deletions were generated based on the results from a manual search for putative NRF1-binding sites in the *SMAD4* promoter region. Significant inhibition of SMAD4 transcriptional activity was observed in HeLa cells upon the ectopic expression of NRF1. Three pairs of promoter regions were derived from a minimal proximal promoter region (−371 bp to −41 bp) of *SMAD4* to identify the region necessary for NRF1 binding more specifically.

Interestingly, the construct with the NRF1-binding site deletion (Luc-41) rescued the promoter activity. Therefore, we concluded that this NRF1-binding site (−186 bp to −175 bp) is crucial for the regulation of *SMAD4* promoter activity. Moreover, the ectopic expression of NRF1 significantly decreases *SMAD4* mRNA transcription and moderately reduces the protein expression (Supplementary Figure S4).

In the genome, a heterodimer of two transcription factors can frequently influence other binding partners, which are likely to be common DNA elements. NRF1, as a regulator of diverse E2F targets, controls cell cycle progression [13,27]. In addition, the transcription factor activity of NRF1 is highly active in human cancer. Recently, Bhawe et al. reported that aberrant NRF1 activity and its regulated genes, including TGF-β1 and p15INK4b, are overexpressed in glioblastoma (high-grade astrocytoma) [15]. p15INK4b, an E2F target gene, also functions as a downstream effector of SMAD4-mediated antiproliferative effects. This prompted us to hypothesize that p15INK4b is a common downstream target of NRF1 and SMAD4. SMAD complexes can occupy the SMAD-binding region (SBR) element located in the distal region of the *p15INK4b* promoter and influence the transcriptional activation of p15INK4b in response to TGF-β [28,29]. Our promoter analysis identified one putative NRF1-binding site (GGCGCATGCGTC) residing approximately 240 bp downstream of the SBR2 element on the *p15INK4b* promoter (Supplementary Figure S5). Although this site is not the perfect palindrome ((T/C)GCGCA(T/C)GCGC(A/G)) usually required for NRF1 recognition, the sequence GCGCRYGCGY is an alternative binding element consensus sequence that is preferred by the chicken NRF1 homolog initiation binding receptor (IBR) [14]. Our ChIP assays confirmed the binding sites of both NRF1 and SMAD4 in the *p15INK4b* promoter region. NRF1 regulates p15INK4b transcription in two manners: (1) When NRF1 is ectopically expressed in the presence of SMAD4, it dramatically decreases the transcription of p15INK4b, regardless of the SBE function (Figure 4B). This suggests that NRF1 directly interacts with the *p15INK4b* promoter either alone or as an NRF1–SMAD4 heterodimer. (2) Alternatively, without a functional NBE, NRF1 can minimize the promoter activity in the presence of SMAD4, indicating an indirect influence likely relying on the NRF1 regulation of SMAD4. In the former scenario, the DNA may form a bent structure, as observed for the binding of transcription regulators, including E2F, to the promoter region [13]. Cyclin D1 and Cyclin D1-associated kinases, whose kinase activity can be inhibited by p15INK4b [30], interact directly with NRF1 in the breast cancer cell line MCF-7 and inhibit NRF1 through phosphorylation [31].

**Figure 6:**
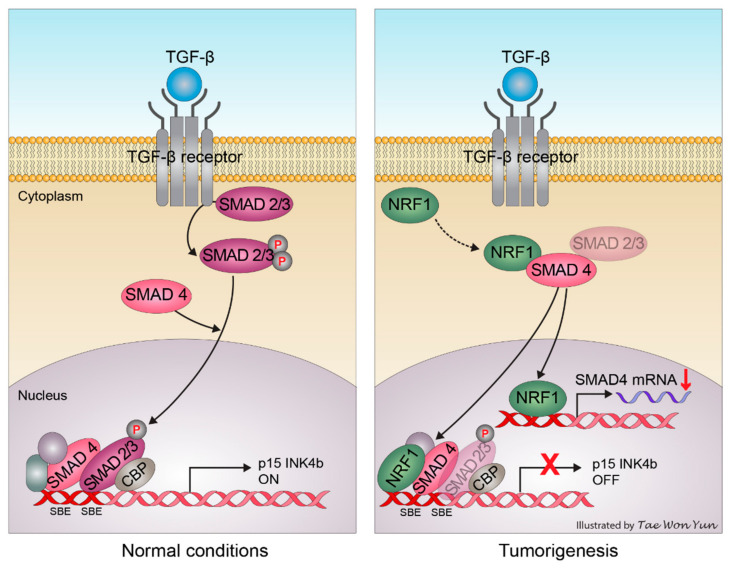
A schematic that depicts the role of NRF1 in the modulation of TGF-β and SMAD4–mediated signal transduction pathways and its impact on the survival of cancer cells.

Together with our findings, this suggests that NRF1 is involved in the regulation of p15INK4b transcription via direct or indirect interactions with SMAD4 (Figure 6). In the absence of NRF1, SMAD4 stimulates p15INK4b transcription through the recruitment of a SMAD protein complex; however, the presence of NRF1 in the *p15INK4b* promoter region prevents p15INK4b induction by negatively regulating SMAD4 transcription. Furthermore, the loss of p15INK4b and SMAD4 activity, mediated by NRF1, can induce an alternative TGF-β signaling pathway in cancer cells, thereby promoting proliferation and tumor progression. Cyclin D1 abundance has been proposed to correlate with the reduced mitochondrial activity due to the reciprocal regulation of NRF1 by Cyclin D1 [31]. Consequently, this decrease in the mitochondrial function might be responsible for the preference of glycolysis over the TCA cycle in cancer cells, a phenomenon named the Warburg effect [32]. Earlier reports of the gene module analysis showed an association between increased nuclear mitochondrial genes, rather than the presence of NRF1 itself, and a poor survival prognosis in breast cancer patients [33]. NRF1, as a hub for p15INK4b regulation, is essential in oncogenesis through its crosstalk with SMAD4 in the TGF-β signaling pathway. To understand the NRF1 and SMAD4 protein–protein interaction more completely, an extensive analysis of each protein and/or structural biology studies will be required.

Recently, Barbagallo, D et al. and Broggi, G et al. reported that the RNA-binding motif responsible for the interaction between Serine and Arginine Rich Splicing Factor 1 (SRSF1) and circSMARCCA5 (a 269-nucleotide-long circRNA) are coupled with increased amounts of total Vascular Endothelial Growth Factor A (*VEGFA*) mRNA secretion and the recommended use of SRSF1 as a diagnostic immunomarker in gliomas [34,35]. In the case of NRF1, it appears to be both a negative regulator and an interaction partner of SMAD4. As such, by forming a heterodimer with SMAD4, NRF1 influences the transcriptional activity of *p15INK4b* by interfering with SMAD4 expression, with a possible dependence on direct contact with the NBE in the *p15INK4b* promoter region. This novel regulation mechanism might be an indicator of the oncogenic role of NRF1. Additionally, the functional loss of SMAD4, caused by an NRF1-induced decrease in p15INK4b transcriptional induction, provides a rationale for the absence of SMAD4 mutations or deletions in some tumor types.

## 4. Materials and Methods

### 4.1. Cell Culture, Transient Transfection, and Treatments

HeLa, SiHa, and MCF7 cells were purchased from the Korean Cell Line Bank (KCLB) and were authenticated by a short tandem repeat (STR) analysis at the cell line core facility at Abion (Abion, Inc., Seoul, Korea). HeLa and SiHa cells were cultured using RPMI 1640, and Dulbecco’s modified Eagle’s medium was used for MCF7 cells. Culture medium was supplemented with 10% fetal bovine serum (Gibco, ThermoFischer Scientific, Seoul, Korea) and 1% penicillin/streptomycin (Gibco, ThermoFischer Scientific, Seoul, Korea). Cells were incubated at 37 °C at 5% CO_2_ atmosphere and were regularly tested for mycoplasma infection using the Myco VALiD Mycoplasma PCR Detection Kit (Intron Biotechnology, Kyung-Gi, Korea). Cultured cells were transiently transfected using Genefectine Reagent (Genetrone Biotech, Gyeonggi-do, Korea). TGF-β1 plasmid (Komabiotech, Seoul, Korea) was transfected at 10 ng/mL.

### 4.2. Western Blotting Analysis

Cells were lysed 24 h post-transfection in RIPA buffer (150-mM NaCl, 10-mM Tris (pH 7.2), 0.1% sodium dodecyl sulphate (SDS), 1% Triton X-100, 1% sodium deoxycholate, and 5-mM ethylenediaminetetraacetic acid (EDTA)) supplemented with protease inhibitor 1 and a phosphatase inhibitor cocktail tablet (Roche Diagnostics, Basel, Switzerland). The cell lysate was incubated at 4 °C for 20 min, followed by centrifugation at 12,000× *g* at 4 °C for 15 min. The amount of protein in each sample was measured using a bicinchoninic acid assay (BCA) kit (Pierce Biotechnology, Rockford, IL, USA). To denature the protein, samples were boiled with SDS sample buffer for 10 min and resolved on 7–10% SDS-polyacrylamide gels and were then transferred onto polyvinyl difluoride (PVDF) membranes. Membranes were blocked with 5% nonfat dry milk in Tris-buffered saline with 0.05% Tween 20 (TBST) to avoid nonspecific antibody binding, followed by incubation with a primary antibody and hybridization with horseradish peroxidase (HRP)-tagged secondary antibody. The primary antibodies SMAD4 (sc-7966), NRF-1 (sc-101102), anti-HA (sc-7392), anti-FLAG (sc-166355), and β-Actin (sc-47778) were purchased from Santa Cruz Biotechnology (Dallas, TX, USA); NRF-1 (ab34682) was purchased from Abcam (Cambridge, MA, USA).

### 4.3. Immunoprecipitation (IP) Assay

HeLa cells were lysed in immunoprecipitation buffer (150-mM NaCl, 50-mM Tris (pH 7.4), 0.5% sodium deoxycholate, and 1% Triton X-100) supplemented with protease inhibitor phosphatase inhibitor cocktail tablets. The antibodies used were anti-SMAD4 (sc-7966), anti-FLAG (sc-166355), anti-MYC (sc-42), and anti-p-SMAD2/3 (sc-11769). After antibody incubation, the lysate was incubated with protein A/G agarose beads (Life Technologies, Carlsbad, CA, USA) overnight at 4 °C. The protein complexes were collected by centrifugation, and equal concentrations of protein samples were separated by SDS-PAGE. Membranes were blocked using 5% skim milk in TBST and were then incubated overnight at 4 °C with primary antibodies (anti-SMAD4 (sc-7966), anti-HA (sc-7392), anti-FLAG (sc-166355), Myc (sc-42), anti-NRF1 (sc-101102), or β-Actin (sc-47778)), followed by incubation with HRP-conjugated secondary antibodies (Pierce Biotechnology, Rockford, IL, USA). The protein bands were visualized with ECL reagent.

### 4.4. Bimolecular Fluorescence Complementation (BiFC) Analysis

BiFC constructs were a kindhearted contribution from Professor Chang-Deng Hu (Department of Medicinal Chemistry and Molecular Pharmacology and Purdue Cancer Center, Purdue University, West Lafayette, IN, USA). cDNAs encoding the NRF1 full sequence or various truncations, amino acid residues 1–108 (NRF 1–108), 108–304 (NRF 108–304), and 304–503 (NRF 304–503), of NRF1, were amplified by PCR from a human cDNA library and subcloned into a pFLAG-CMV vector to generate BiFC fusion constructs with N--terminal nonfluorescent fragments of Venus (VN173). cDNAs encoding SMAD4, SMAD4-MH1 (residues 1–145), SMAD4-MH2 (residues 321–553), and SMAD4-linker (residues 140–325) were amplified by PCR from a human cDNA library and subcloned into a pHA-CMV vector to generate BiFC fusion constructs with C-terminal nonfluorescent fragments of Venus (VC155). The BiFC analysis was performed as previously described [24].

### 4.5. The Proximity Ligation (PLA) Assay

HeLa cells were used for the PLA experiments following the manufacturer’s protocol (O-LINK Bioscience, Uppsala, Sweden). Paraformaldehyde fixed cells on a 4-well cell culture slide (SPL Life Sciences Co., Gyeonggi-do, Korea) by the overnight incubation of were incubated with NRF1 and SMAD4 antibodies at 4 °C overnight, after 1 h of blocking with 5% nonfat milk to avoid nonspecific binding. Cells were washed twice with TBST for 5 min and incubated with the PLA probe solutions for 60 min at 37 °C. After incubation, cells were washed with TBST for 5 min and incubated for half an hour with the ligase solution at 37 °C. Samples were washed twice with TBST for 2 min, followed by amplification using polymerase solution for 100 min at 37 °C. The samples were washed with SSC buffers (prepared according to the manufacturer’s recipe) before mounting [24]. The slides were visualized, and images were taken using an LSM 700 ZEISS laser scanning confocal microscope (Carl Zeiss, Jena, Germany).

### 4.6. Quantitative Real-Time Polymerase Chain Reaction (qRT-PCR) Analysis

Total RNA was extracted from transfected cells using the Hybrid-RTM total RNA Kit (GeneAll Biotechnology, Seoul, Korea). Following cDNA synthesis using the Superscript II First-Strand Synthesis System (Life Technologies), qRT-PCR was executed with a dual system LightCycler (Roche Diagnostics). The SYBR Green-based comparative CT method was used to analyze the target gene expression in relation to HPRT expression (relative fold-change = 2^−ΔΔCT^) [36]. Primers used are listed in Supplementary Table S1. All PCR primers were purchased from Cosmo Genetech (Seoul, Korea).

### 4.7. Luciferase Reporter Gene Assay

Luciferase reporter constructs were cloned using the restriction map of the BAC729G3 bacterial artificial chromosome (BAC) clone from the RPCI-11 human BAC library (Invitrogen, Carlsbad, CA, USA), which covers the alternative promoter region of SMAD4 (Luc-371) [24,36]. All SMAD4 constructs (Luc-371, Luc-216, and Luc-41) were confirmed using sequencing. Twenty-four hours after the transfection, cells were lysed with luciferase assay buffer. Luciferase activity was measured using the dual-luciferase reporter assay system following the manufacturer’s protocols (Promega, Madison, WI, USA), and luminescence was measured using a GENios Pro Microplate Reader (Tecan Trading AG, Mannedorf, Switzerland).

### 4.8. Chromatin Immunoprecipitation (ChIP) Assays

ChIP assays were performed using the EZ-ChIP Kit (Millipore, Bedford, MA, USA). The immunoprecipitation antibodies anti-SMAD4 (sc-7966), mouse IgG, and rabbit IgG were purchased from Santa Cruz Biotechnology, and pSMAD2 (Ser 465/467)/SMAD3 (Ser423/425) [#8828] was purchased from Cell Signaling Technology (Danvers, MA, USA). Conventional PCR methods were used to confirm the *SMAD4* promoter-specific regions using the respective primers: SMAD4 ChIP I (Forward) 5′-TGC CTA CGC AGG TCC TCA -3′ and (Reverse) 5′-GCG GCT CTG AAT CTG GAC -3′ and SMAD4 ChIP II (Forward) 5′-GTC CAG ATT CAG AGC CGC-3′ and (Reverse) 5′-CCA AAC CGC TCC GTT ACC -3′. A distal region of the *p15INK4b* promoter (nucleotides -547 to -239) was amplified using the following primers: 5′-TAT GGT TGA CTA ATT CAA ACA-3′ (Forward) and 5′-AAT ATT TTG GGA ATG TTC ACC A -3′ (Reverse) [36,37]. In the proximal region of the *p15INK4b* promoter (nucleotides -47 to 161), a 208-bp segment was amplified using the following primers: 5′-GCC CCT TGG CCC AGC TGA AAA C-3′ (Forward) and 5′-TTA GCT CCG GGC TTT TCC TGG C-3′ (Reverse). PCR amplification was performed for 30 cycles at 94 °C (30 s), 60 °C (30 s), and 72 °C (30 s).

### 4.9. Screening Analysis of Transcription Factor Binding Sites (TFBS)

Screening of TFBS was carried out as described previously [24,36]. Briefly, regulatory elements in the core promoter region were identified using MatInspector (Genomatix Software GmbH, Munich, Germany; http://www.genomatix.de, accessed on 1 January 2010). The TFBS program (http://www.cbrc.jp/research/db/TFSEARCH.html, accessed on 1 January 2010) was used to identify the putative NRF1 transcription factor binding sites within the 5′-flanking region of the *SMAD4* promoter.

### 4.10. Statistical Analysis

The results were statistically validated using one-way ANOVA analysis followed by Tukey’s test for multiple comparisons. GraphPad prism software was used for all statistical analyses (GraphPad Software Inc., San Diego, CA, USA). Results were considered significant at * *p* < 0.05, ** *p* < 0.001, *** *p* < 0.001, or **** *p* < 0.0001. All the data with error bars represent means ± SD for at least three independent experiments.

## Figures and Tables

**Table 1:** List of putative SMAD4-binding partners.

Gene Name	Protein Name	Z-Score	Molecular Function
CCDC149	Coiled-coil domain-containing protein 149	19.86227	none
TFEB	Transcription factor EB	13.14539	Transcription factor activity
FAM64A	Protein FAM64A	11.54105	none
VASH2	Vasohibin-2	9.51351	Positive regulation of Angiogenesis
ABT1	Activator of basal transcription 1	8.0119	Transcription factor activity
ROBO3	ROBO3 protein	7.69706	Neuron migration
AEBP2	Zinc finger protein AEBP2	6.16836	Transcription factor activity
RNPC3	RNA-binding protein 40	5.36746	RNA splicing
TFE3	Transcription factor E3	5.11651	Transcription factor activity
AURKA	Aurora kinase A	4.55737	Positive regulation of mitosis
PHF7	PHD finger protein 7	4.38257	Zinc ion binding
ARHGAP15	Rho GTPase-activating protein 15	4.14593	small GTPase mediated signal transduction
MITF	Microphthalmia-associated transcription factor	4.07745	Multicellular organismal development
COIL	Coilin	3.99107	disulfide oxidoreductase activity
RAB24	Ras-related protein Rab-24	3.43295	small GTPase mediated signal transduction
ARHGEF5	Rho guanine nucleotide exchange factor 5	3.3522	Regulation of Rho GTPase activity
UCHL3	Ubiquitin carboxyl-terminal hydrolase isozyme L3	3.27706	Ubiquitin-dependent protein catabolic process
IER3	Radiation-inducible immediate-early gene IEX-1	3.0348	Positive regulation of apoptotic process
EIF2S2	EIF2S2 protein	2.97858	Translation initiation factor activity
NRF1	Nuclear respiratory factor 1	2.87892	Generation of precursor metabolites and energy
ANXA10	Annexin A10	2.53086	Calcium-dependent phospholipid binding
FN1	FN1 protein	2.52421	None
SENP8	Sentrin-specific protease 8	2.40973	Cysteine-type peptidase activity
GNAI2	Guanine nucleotide-binding protein G(i) subunit alpha-2	2.37446	Signal transducer activity
ITK	Tyrosine-protein kinase ITK/TSK	2.15929	ATP binding
MMAB	Methylmalonic aciduria type B protein	2.11635	cob(I)yrinic acid a,c-diamide adenosyltransferase activity

## Data Availability

Not applicable.

## Supplementary Materials

The following are available online at https://www.mdpi.com/article/10.3390/ijms22115595/s1. References [3,24] are cited in the supplementary materials.
