# Supplementary material for: Nuclear Respiratory Factor-1, a Novel SMAD4 Binding Protein, Represses TGF-β/SMAD4 Signaling by Functioning as a Transcriptional Cofactor
Source: Int J Mol Sci. 2021 May 25;22(11):5595. doi: 10.3390/ijms22115595 (PMC8198518; doi:10.3390/ijms22115595)
Supplement: Supplementary file 1 [file ijms-22-05595-s001.zip › ijms-1227248-supplementary.pdf]

Article

Nuclear Respiratory Factor-1, a Novel SMAD4 Binding Protein, Represses TGF-β/SMAD4 Signaling by Functioning as A Transcriptional Cofactor

Nirmal Rajasekaran [1,†], Kyoung Song [2,†], Jin-Hee Lee [1], Yun Wei [1], Özgür Cem Erkin [3], Hunseok Lee [1] and Young Kee Shin [1,4,5,*]

1 Laboratory of Molecular Pathology and Cancer Genomics, Research Institute of Pharmaceutical Sciences and College of Pharmacy, Seoul National University, Seoul 08826, Korea; nirmalpharma@gmail.com (N.R.); jinheelee80@gmail.com (J.-H.L.); yunwei.bio@gmail.com (Y.W.); ryanlee0@snu.ac.kr (H.L.)
2 College of Pharmacy, Duksung Women's University, Seoul 01369, Korea; songseoul17@duksung.ac.kr
3 Department of Bioengineering, Faculty of Engineering, Adana Science and Technology, Adana 01250, Turkey; ocerkin@yahoo.com
4 Department of Molecular Medicine and Biopharmaceutical Sciences, Graduate School of Convergence
Science and Technology, Seoul National University, Seoul 08826, Korea
5 Bio-MAX, Seoul National University, Seoul 08826, Korea
* Correspondence: ykeeshin@snu.ac.kr; Tel.: +82-2-880-9126; Fax: +82-2-883-9126
† These authors contributed equally to this work.

Supplementary Materials and Methods

1.1 Competition bimolecular fluorescence complementation (BiFC) assays

For the BiFC assays, HeLa cells were transiently co-transfected with 0.5 μg Flag-*VN173*–tagged NRF1 domains (full length, NRF 1-108 , NRF 108-304, or NRF 304-503) , 0.5 μg *VC155*-tagged SMAD4 domains (full length, MH1, Linker, or MH2) with or without 0.5 μg of 3xFLAG-tagged full length NRF1. BiFC analysis was performed 24 h post-treatment. The detectable fluorescence signal indicates protein–protein interactions after co-transfection of the VC155 and VN173 plasmids [24]. The fluorescence signals were captured using a charge-coupled device (CCD) camera mounted on a TE2000-U inverted fluorescence microscope (Nikon, Melville, NY) with JP4 filters (Chroma, Rockingham, VT, USA).

1.2 DNA pull-down assays

DNA pull-down assays were performed as previously described [3]. Briefly, 5 μg biotinylated double-stranded oligonucleotides corresponding to the NRF1 nuclear binding elements (NBEs) for the wild-type *SMAD4* promoter (Forward 5′ -CTC CGC CC**G CGC TGA GGC** CCA GGC CC- 3′ and Reverse 3′ -GGG CCT GG**G CCT CAG CGC** GGG CGG AG -5′), and the mutant (Forward 5′ -CTC CGC CC**A TTA TGT AAT** CCA GGC CC- 3′ and Reverse 3′ -GGG CCT GG**A TTA CAT AAT** GGG CGG AG- 5′) were obtained by PCR amplification using synthetic biotinylated primers (Bioneer, Daejeon, Korea). DNA pull-downs were performed using 100 μg total cell lysate (HeLa cells transfected

with 5 μg of 3xFlag-NRF1) in buffer containing 50 mM Tris-HCl, pH 7.5, 10% Glycerol, 140 mM NaCl, 3 mM $MgCl_2$, 0.2 mM EDTA, and 10% Triton X-100; the buffer was supplemented with protease inhibitor at 1:1000 (all from Sigma-Aldrich, St Louis, MO, USA). Due to the affinity of Dynabeads Streptavidin (Dynal M270, Thermo Fischer Scientific Inc,) for biotinylated DNA, the DNA-protein complexes were pulled down with Dynabeads Streptavidin by centrifugation at 200 × g for 60 s. The pulled-down complexes were washed three times with 1 mL ice-cold Tris buffer (50 mM Tris, 100 mM KCl, 1mM EDTA, 5% glycerol, and 1 mM DTT), separated on an SDS-polyacrylamide gel, and visualized using western blot analysis.

The *p15INK4b* DNA pull-down assay was performed similarly using biotinylated oligonucleotides corresponding to the NBE in the WT (Forward 5′ -CAG TCT CT**G GCG CAT GCG TC**C TAG CAT C -3′ and Reverse 3′ -GAT GCT A**GG ACG CAT GCG CC**A GAG ACT G -5′) and mutant (Forward 5′ -CAG TCT CT**G ATT AAT TAT TC**C TAG CAT C -3′ and Reverse 3′ -GAT GCT AGG AAT AAT TAA TCA GAG ACT G -5′) *p15INK4b* promoter.

Supplementary Table S1. List of qRT-PCR primers and probes.

NRF1 (S)	CAG TCA CTA TGG CGC TTA ACA
NRF1 (A)	ATC TGT CCC CCA CCT TGT AA
TaqMan probe (S)	FAM – GGC TGA GG – Dark quencher
SMAD4 (S)	TGG CCC AGG ATC AGT AGG T
SMAD4 (A)	CAT CAA CAC CAA TTC CAG CA
TaqMan probe (S)	FAM - CTC CAG CT – Dark quencher
p15INK4b (S)	CAA CGG AGT CAA CCG TTT C
p15INK4b (A)	GGT GAG AGT GGC AGG GTC T
TaqMan probe (S)	FAM - TCC AGG TC – Dark quencher

Supplementary Figures (S1-S5)

Supplementary Figure S1.

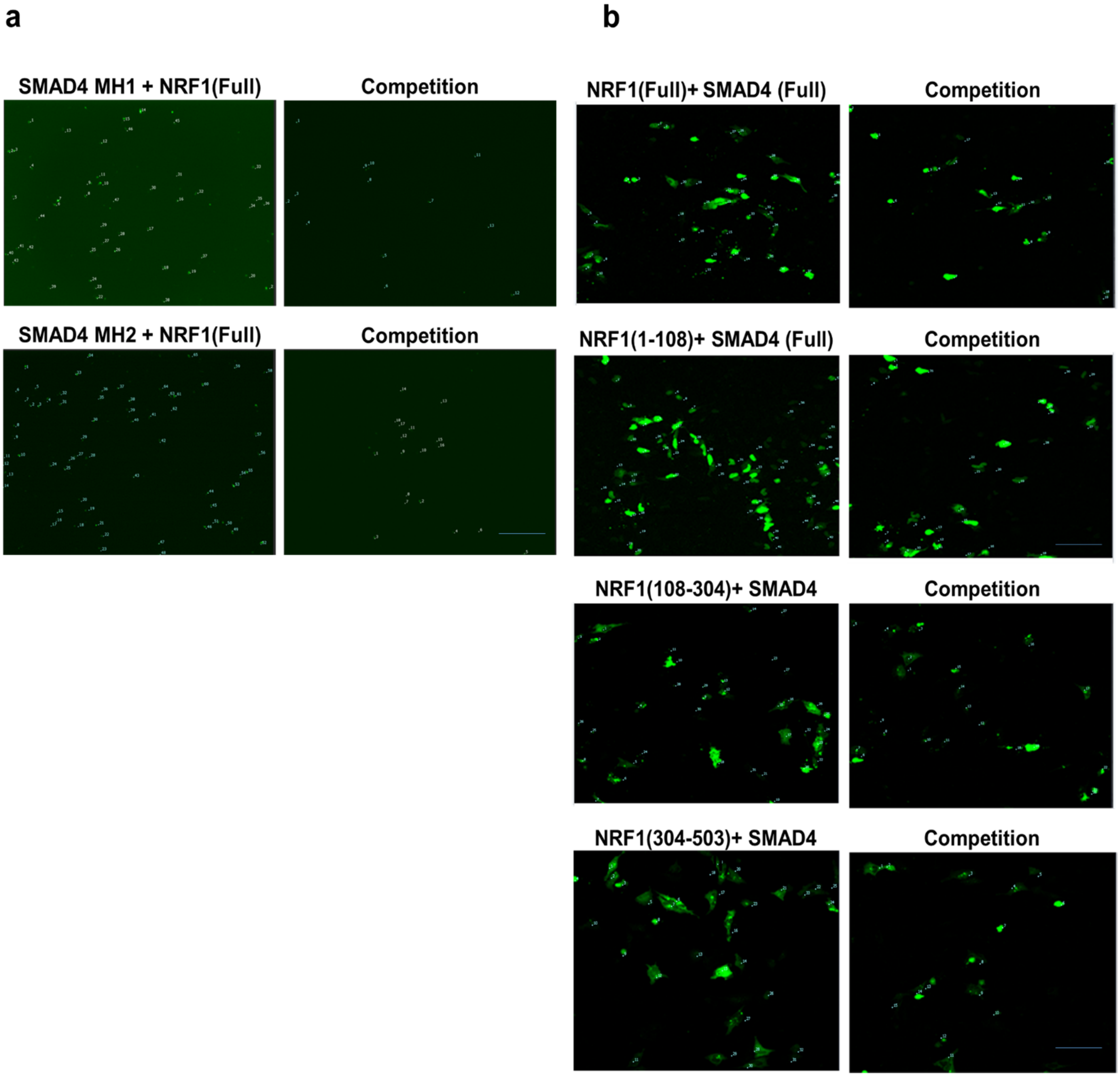

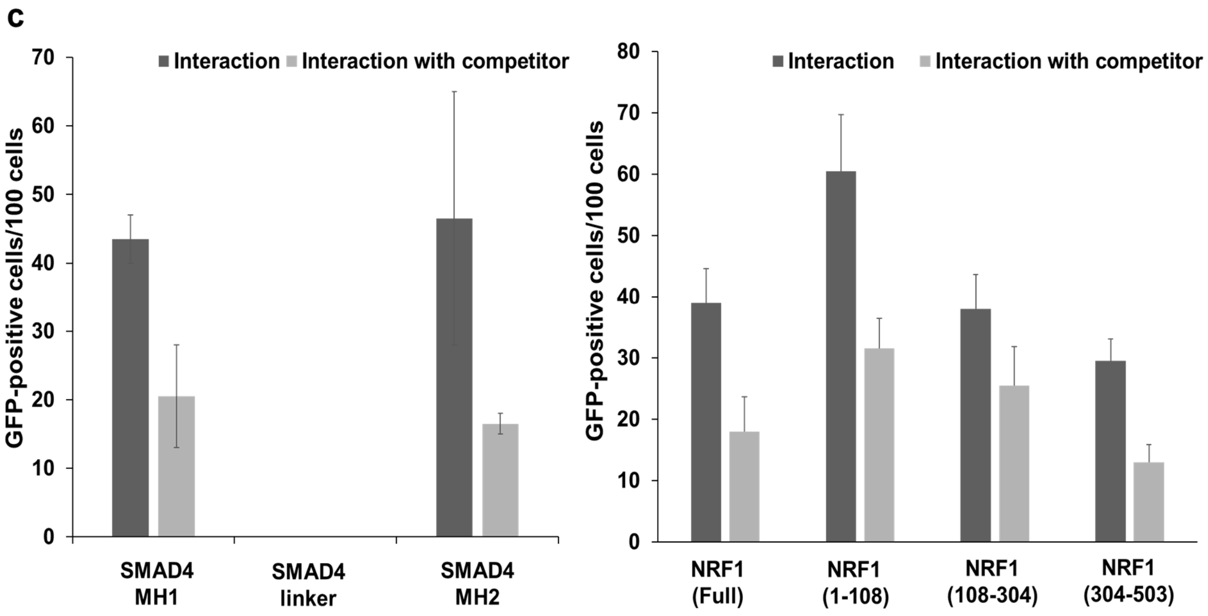

Supplementary Figure S1. Specific binding confirmation using the BiFC competition assay. HeLa cells transiently transfected with **a** *FLAG-VN173*-tagged NRF1 or its truncated constructs and **b** *HA-VC155*-tagged SMAD4 or its truncated constructs with NRF1 and SMAD4 without tagging as competitor. BiFC analysis was performed 24 h post-transfection. **c** for the competition assay, HeLa cells were co-transfected with plasmids encoding FLAG-VN173-tagged NRF1 and HA-VC155-tagged SMAD4 in the absence or presence of co-transfection with the 3xFLAG-tagged NRF1 plasmid (0.5 mg). The fluorescence signal was captured under fluorescence microscopy (x10) and counted in 5 random areas. GFP-positive cells were normalized with total cell counts and represented counts/100 cells. Scale bar, 100 mm. Values are the means ± SD.

Supplementary Figure S2.

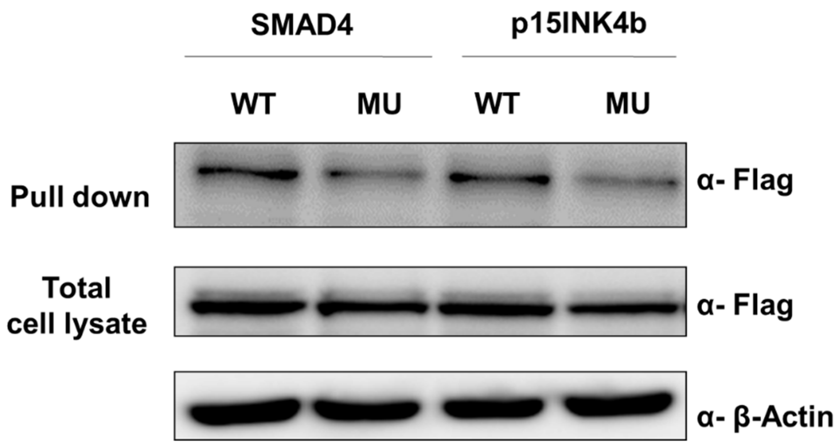

Supplementary Fig S2. DNA pull-down assay. NRF1 can be recruited to the NRF1 binding elements (NBE) located on the *SMAD4* and *p15INK4b* promoters. The binding of 3xFLAG-tagged NRF1 on the NBE of *SMAD4* (WT), *SMAD4* (mutant), *p15INK4b* (WT), and *p15INK4b* (mutant) promoter sequences was confirmed by a DNA pull-down assay. The results indicate that introducing mutations in the NBE affects NRF1 recruitment.

Supplementary Figure S3.

- 432

ctacaacctagccaccttctccccagagctgtcgactggctgttgaaggccaatttttgtgcctacgcaggtcctcaacaca
gaacaaaacaaaaaaacaacaaaggccgggctaatagctatttataaacacttactggacgcccactctacgccgag
ctctcccgcgctccttggatactttttgcaacgagatgccaatttccccggcgaccactccctcaaacaggccttcgcctcc
gccc**gcgctgaggc**ccaggcccaggtccagattcagagccgcccgccggctggcgctgccctgtag**gcgcctgcgc**
agagcgaccctccccgtcactcggagcgggaggcgggggcagccgggagaaaggaaagctgcgggggaaaaggg
ccaaaccctgaaattacccggatgtggtcc**ccgcgcgcgc**at**gctcagtggc**ttctcgacaagttggcagcaac…….

+1 (Transcription start site)

Supplementary Figure S3. NRF1 binding sites within the *SMAD4* promoter region -500bp to the transcription start site was identified using MatInspector professional software and the internet-based TFSEARCH database.

Supplementary Figure S4.

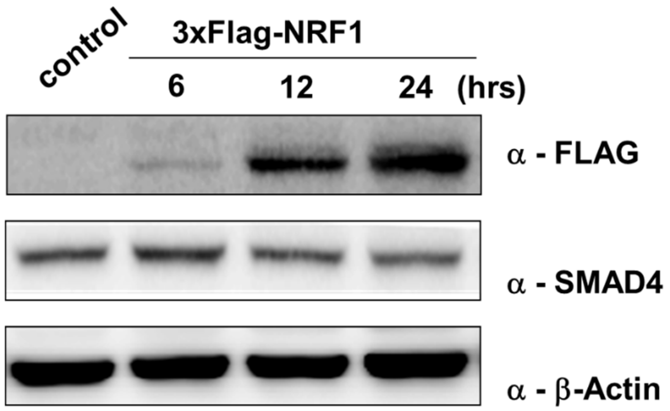

Supplementary Figure S4. SMAD4 expression is affected by NRF1 overexpression. Western blot results show changes in SMAD4 expression with an incremental increase in NRF expression.

Supplementary Figure S5.

```
-716 GGATCTCAGATTCTTAAAGTATAATTTTTTTTTGTCTTATGTGTGCCAGGTTGCCACTCT -657
-656 CAATCTCGAACTAGTTTTTTTTCTCTTTTAAGGTTGTATCCATAATGCAAAAATGGAAAG -597
-596 AATTAAAAAGCACACGCAAAACATGATTCTCGGGATTTTTCTCTATTTTTATGGTTGACT -537
-536 AATTCAAACAGAAAGACACATCCAAGAGAAAATTGCTAAGTTTGATACAAGTTATGAAAC -477
-476 TTGTGAAGCCCAAGTACTGCCTGGGGATGAATTTAACTTGTATGACAGGTGCAGAGCTGT -417
-416 CGCTTTCAGACATCTTAAGAAACACGGAGTTATTTTGAATGACTTTCTCTCGGTCACAAG -357
-356 GGAGCCACCAACGTCTCCACAGTGAAACCAACTGGCTGGCTGAAGGAACAGAAATCCTCT -297
-296 GCTCCGCCTACTGGGGATTAGGAGCTGAGGGCAGTGGTGAACATTCCCAAAATATTAGCC -237
-236 TTGGCTTTACTGGACATCCAGCGAGCAGTGCAGCCAGCATTCCTGGCGGCTCCCTGGCCC -177
-176 AGTCTCTGGCGCATGCGTCCTAGCATCTTTGGGCAGGCTTCCCCGCCCTCGTGACGCGTC -117
-116 GGCCCGGGCCTGGCCTCCCGGCGATCACAGCGGACAGGGGGCGGAGCCTAAGGGGGTGGG  -57
 -56 GAGACGCCGGCCCCTTGGCCCAGCTGAAAACGGAATTCTTTGCCGGCTGGCTCCCCACTC    3
   4 TGCCAGAGCGAGGCGGGGCAGTGAGGACTCCGCGACGCGTCCGCACCCTGCGGCCAGAGC   63
  64 GGCTTTGAGCTCGGCTGCGTCCGCGCTAGGCGCTTTTTCCCAGAAGCAATCCAGGCGCGC  123
 124 CCGCTGGTTCTTGAGCGCCAGGAAAAGCCCGGAGCTAACGACCGGCCGCTCGGCCACTGC  183
```

NRF1 **NT 158-169**

SMAD4 **NT 523-520, 406- 409**

Supplementary Figure S5. The distal region of the p15INK4b promoter. SMAD4 and NRF1 binding sites within the p15INK4b promoter are highlighted as indicated.
